# Environmental control of plant nuclear gene expression by chloroplast redox signals

**DOI:** 10.3389/fpls.2012.00257

**Published:** 2012-11-19

**Authors:** Jeannette Pfalz, Monique Liebers, Matthias Hirth, Björn Grübler, Ute Holtzegel, Yvonne Schröter, Lars Dietzel, Thomas Pfannschmidt

**Affiliations:** Junior Research Group “Plant acclimation to environmental changes: Protein analysis by MS,” Department of Plant Physiology, Institute of General Botany and Plant Physiology, Friedrich-Schiller-University JenaJena, Germany

**Keywords:** photosynthetic acclimation, electron transport, redox signaling, gene expression, environmental sensing

## Abstract

Plant photosynthesis takes place in specialized cell organelles, the chloroplasts, which perform all essential steps of this process. The proteins involved in photosynthesis are encoded by genes located on the plastid and nuclear genomes. Proper function and regulation of light harvesting and energy fixation thus requires a tight coordination of the gene expression machineries in the two genetic compartments. This is achieved by a bi-directional exchange of information between nucleus and plastids. Signals emerging from plastids report the functional and developmental state of the organelle to the nucleus and initiate distinct nuclear gene expression profiles, which trigger responses that support or improve plastid functions. Recent research indicated that this signaling is absolutely essential for plant growth and development. Reduction/oxidation (redox) signals from photosynthesis are key players in this information network since they do report functional disturbances in photosynthesis, the primary energy source of plants. Such disturbances are caused by environmental fluctuations for instance in illumination, temperature, or water availability. These environmental changes affect the linear electron flow of photosynthesis and result in changes of the redox state of the components involved [e.g., the plastoquinone (PQ) pool] or coupled to it (e.g., the thioredoxin pool). Thus, the changes in redox state directly reflect the environmental impact and serve as immediate plastidial signals to the nucleus. The triggered responses range from counterbalancing reactions within the physiological range up to severe stress responses including cell death. This review focuses on physiological redox signals from photosynthetic electron transport (PET), their relation to the environment, potential transduction pathways to the nucleus and their impact on nuclear gene expression.

## Introduction

Plants are sessile and, therefore, cannot escape from unfavorable conditions in their environment. During evolution they developed a number of responses which help them to deal with varying and adverse environmental cues. These responses cover several time scales acting at the physiological level within minutes, at the developmental level within days and at the seasonal level within months. Most of these responses work at the molecular level including regulation of gene expression networks, adjustment of metabolic pathways, or nutrient allocation. One important sensing system of plants for changes in the environment is photosynthesis. Its unique combination of light-dependent light harvesting processes and temperature-dependent carbon fixation reactions makes it ideal for precise and rapid detection of abiotic environmental changes since for optimal photosynthesis both parts needs to work in a fine-tuned balance. Changes in temperature, light intensity or quality, or in the availability of water, CO_2_, or nutrients may disturb this balance resulting in less efficient photosynthesis. In all cases the immediate effect is a change in photosynthetic electron flux which affects the reduction/oxidation (redox) state of the components involved in it. In many cases this change in redox state initiates acclimation responses which help the plant adapting the photosynthetic process to the changed environment (Anderson et al., 1995; Kanervo et al., 2005; Walters, 2005; Dietzel et al., 2008a; Eberhard et al., 2008). However, since photosynthesis is the ultimate source of energy for plants it is tightly connected with many other physiological and metabolic processes. The redox signals regulating photosynthesis, thus, lead to a systemic response also in non-photosynthetic processes. It is important to note that the type of response depends highly on strength and duration of the environmental disturbance and its effect on photosynthesis. In recent years laboratory experiments have uncovered a number of strategies how plants cope with the environment, but we are far away from understanding these responses under free-fluctuating conditions in nature which can vary in an unpredictable way. It becomes increasingly clear that many redox signals occur at the same time or in varying combinations when observed under natural or variable experimental conditions. It is, therefore, reasonable to assume the action of redox signaling networks rather than that of single signaling pathways. Nevertheless, for building networks it is essential to understand the immediate molecular mechanisms initiated by a distinct redox signal. Thus, in future a combined strategy of experiments with a single changing parameter and experiments with two or more changing parameters will be required.

## Important operational redox signals from photosynthesis

Photosynthesis, in simple terms, is the light-driven transfer of electrons and protons from water to NADP^+^, the formation of ATP using the trans-thylakoidal proton gradient generated during this transfer and, subsequently, the use of these reduction and energy equivalents in the fixation of CO_2_ to produce carbohydrates as chemical energy source for growth and development of the organism (Buchanan et al., 2002). This complex process contains many reduction and oxidation steps and, therefore, the components involved change their redox status depending on the efficiency of photosynthesis. Two of them, plastoquinone (PQ) and thioredoxin, are of special importance as they fulfil not only a function as redox-active molecules but also initiate signaling cascades which control molecular responses acclimating photosynthesis to the environment (Aro and Andersson, 2001; Foyer et al., 2012). This includes also the control of nuclear gene expression and, thus, represents an example for operational control in retrograde signaling (Pogson et al., 2008) which will be the focus of this review.

PQ is the intermediate electron carrier which connects photosystem (PS) II and the cytochrome (cyt) b_6_f complex in the photosynthetic electron transport (PET) chain. This location makes this molecule pool very sensitive to any imbalance in the relative activities of PSII and PSI, especially since the PQ oxidation represents the slowest step in linear electron transport (Allen, 2004). Under conditions favoring PSII PSI becomes rate-limiting and the PQ pool receives more electrons from PSII than it can deliver to PSI resulting in a reduction of PQ. Under conditions favoring PSI the opposite situation is established and the pool becomes oxidized. These redox changes occur almost immediately and can be induced either physiologically by environmental changes or chemically by treatment with electron transport inhibitors (Pfannschmidt et al., 2009). DCMU irreversibly binds to the Q_B_ binding site of PSII and prevents any electron transport from PSII to subsequent acceptors resulting in oxidation of the PQ pool. DBMIB binds to the plastoquinol-oxidation site of the cyt b_6_f complex resulting in PQ reduction (Trebst, 1980). It must be noted that DBMIB is a labile compound which becomes easily inactivated if not re-supplied. Furthermore it can loose its specificity since at high concentrations it also binds to the DCMU binding site. Experiments based on such inhibitor treatments, therefore, essentially require titration controls and must be checked for side effects (Pfannschmidt et al., 2009).

In photosynthesis the PQ redox state controls phosphorylation of light harvesting complex proteins of PSII (LHCII) and the relative allocation of the mobile antenna to the two PS. This short-term response (half-time ~10 min) balances excitation energy distribution between the PSs (state transitions) and requires the action of the thylakoid-bound kinase STN7 (Rochaix, 2007). In the long-term (half-time 1–2 days) PQ redox state controls the adjustment of PS stoichiometry which requires a tight control of both plastid and nuclear encoded photosynthesis genes (Allen and Pfannschmidt, 2000). The latter requires the transduction of the PQ redox signal toward the nuclear compartment and represents an important plastidial signal.

Thioredoxins are a family of small proteins with a size of ~12 kDa which possess a redox-active dithiol group in a conserved WCGPC amino acid motif (Schurmann and Buchanan, 2008). In *Arabidopsis* 44 different thioredoxins have been identified and a large number of them are active in the chloroplast (Meyer et al., 2005). These receive their electrons from PSI *via* the action of an enzyme called ferredoxin–thioredoxin oxidoreductase (FTR). In the dark thioredoxins are usually oxidized but become rapidly reduced upon illumination when the PET chain is activated. In their reduced state they are able to reduce regulatory thiol groups especially in the enzymes of the Calvin–Benson cycle and, by this means, control production of carbohydrates in the carbon reduction cycle of photosynthesis. They also functionally separate the reductive and oxidative pentose-phosphate pathway avoiding futile cycling of common substrates (Buchanan et al., 2002).

In recent years a number of additional thioredoxin targets have been identified for instance in the lumen (Buchanan and Balmer, 2005; Buchanan and Luan, 2005). Most recently a novel thioredoxin-like protein designated as TrxZ has been identified as subunit of the plastid RNA polymerase complex potentially linking redox regulation and plastid transcription (Arsova et al., 2010; Schroter et al., 2010). Inactivation of its gene in *Arabidopsis* creates an albino phenotype indicating that it cannot be replaced by any other thioredoxin, a property which is unique in this group of reductive proteins.

As an unavoidable side reaction of oxygenic photosynthesis reaction centres transfer electrons not only to their primary acceptors but also to molecular oxygen in their immediate surrounding generating reactive oxygen species (ROS) such as superoxide (mainly at PSI *via* ferredoxin) or singlet oxygen (mainly at PSII *via* triplett state chlorophylls) (Apel and Hirt, 2004). ROS induce oxidative damages to proteins or membrane lipids and, therefore, are harmful for all kinds of biological molecules including the photosynthetic apparatus itself (e.g., during photoinhibition). Plant cells and chloroplasts, therefore, possess a sophisticated antioxidant network consisting of reductive low-molecular weight components (glutathione, ascorbate, and α-tocopherol) (Szarka et al., 2012) as well as various enzymatic activities (superoxide dismutases, ascorbate peroxidises, peroxiredoxins, and glutaredoxins) (Dietz, 2011; Zaffagnini et al., 2012) which scavenge and detoxify ROS and a number of recently discovered reactive nitrogen species (NOS) such as nitric oxide (NO) (Navrot et al., 2011). However, ROS and NOS possess a dual role and act also as important signaling molecules in stress responses including pathogen defense and programmed cell death. This includes a number of redox-dependent protein modifications such as glutathionylation or nitrosylation. This area of research is rapidly expanding and has been extensively reviewed elsewhere. This review will include only that work which is essential for understanding of physiological redox signals from the PQ and Trx pools and the reader interested in details of stress responses is referred to the reviews mentioned above and the references therein.

## Environmental induction of photosynthetic redox signals

**Figure 1:**
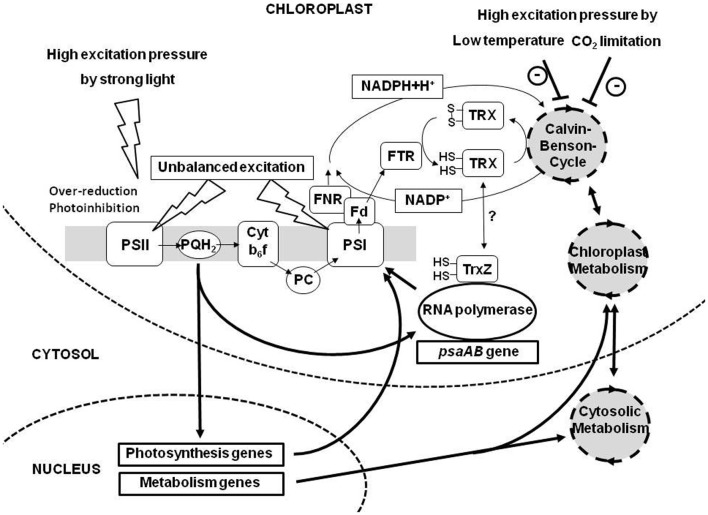
**Overview of environmental constraints affecting photosynthesis and redox-controlled gene expression events.** Three major compartments of plant cells, chloroplast, cytosol, and nucleus are shown. The photosynthetic electron transport chain (consisting of the complexes PSII, Cyt b_6_f complex, and PSI) and the coupled carbon fixation reactions are depicted schematically. Redox signals from the PQ pool (PQH2) and the thioredoxin system (TRX) are induced by changes in light, temperature, and CO_2_ availability or combinations of these parameters. Electron transport and transfer of reducing power are indicated by thin black arrows. Redox signaling, metabolic interactions, and feedback regulation from gene expression are indicated by thick black arrows. Fd, ferredoxin; FNR, ferredoxin-NADP-oxidoreductase; FTR, ferredoxin-thioredoxin-oxidoreductase; PC, plastocyanin; *psaAB*, plastid genes for the apoproteins PsaA and PsaB of PSI (the main triggers of photosystem stoichiometry adjustment). For further details see text. How TrxZ is reduced and how it regulates the plastid RNA polymerase is unknown to date.

The processes of light energy absorption, transfer within the antenna, and charge separation depend mainly on the biophysical properties of the reaction centres and their antenna structure. These processes are largely independent from ambient temperature; however, the amounts of photons absorbed and the efficiency of their transport are directly influenced by the quantity and the quality of the incident light (see also below). In addition, in natural habitats the illumination of plants is not constant but fluctuates within seconds and minutes, as well as daily and seasonal periods, having multiple effects on electron transport efficiency. In contrast, subsequent metabolic reaction such as the Calvin–Benson cycle, N- and S-reduction reactions or other plastid biosynthesis pathways are not directly affected by light but are strongly influenced by the environmental temperature as well as by availability of substrates, nutrients, carbon, or water. Nevertheless they require sufficient amounts of energy and reduction equivalents (i.e., ATP and NADPH_2_) from the light reaction for optimal activity. Thus they are also dependent on the functionality of PET *via* its production of ATP and NADPH_2_. On the other hand efficient PET is possible only if a sufficiently high concentration of final electron acceptors (i.e., NADP^+^) is available. If metabolic activities and, thus, the use of ATP and NADPH_2_ are down-regulated (for instance by low temperatures), the availability of these final acceptors can become limiting leading to a feedback restriction in electron transport efficiency. In conclusion, there exists a delicate balance between photosynthetic light reaction and subsequent metabolism leading to a mutual dependency which is largely determined by the residing environmental condition (Figure 1). This makes photosynthesis a very sensitive system for environmental changes in the surrounding of the plant which are reflected by the reduction state of the electron transport chain, i.e., the PQ pool and the thioredoxin system.

Well known and characterized in this context are the influences of excitation pressure on PSII (Figure 1). Under conditions of high excitation pressure absorbed light energy exceeds the demands of the dark reaction leading to a reduction of the electron transport chain while under conditions of low excitation pressure the opposite occurs (Hüner et al., 1998). These two situations are, however, not unambiguous with respect to the inducing environmental condition. High excitation pressure can be induced by sudden and strong increases in light intensity resulting in the absorption of too much photons (Karpinski et al., 1997), but it can be also induced by much weaker light intensities when the efficiency of the dark reaction is strongly restricted by, e.g., a shift to low temperatures or low water availability (Ensminger et al., 2006). All situations will result in a reduced PQ pool and the increased formation of ROS (as well as a number of other signals not clearly defined yet) exacerbating a clear interpretation and understanding of results from experiments under free running conditions. Experimental set-ups, therefore, are usually designed in a way that only one environmental parameter is changed in a distinct manner to understand the respective signaling and the induced acclimation responses. Very common are variations in light intensity at a constant temperature, but also variations of temperature at a constant light intensity are useful to study the effects of excitation pressure (Hüner et al., 1998). Alternatively the redox state of the electron transport chain can be manipulated by growing plants under artificial light sources with varying light quality (Chow et al., 1990; Melis, 1991; Allen, 1992). This set-up uses the fact that the absorption maxima of PSII and PSI differ by 20 nm (680 versus 700 nm). In natural habitats a strong enrichment of far-red wave lengths occurs within a canopy or dense plant population due to selective absorption of blue and red wavelengths in the top leaf layer (Terashima and Hikosaka, 1995; Smith, 2000; Dietzel et al., 2008a). This leads to an over-excitation of PSI relative to PSII and to an imbalance in excitation energy distribution between the PSs (Figure 1) which is counteracted by state transitions or PS stoichiometry adjustment. Under laboratory conditions this effect can be mimicked with the use of special light sources which excite preferentially PSII or PSI leading to a reduction or oxidation of the electron transport chain. An advantage of this system is that it works in a weak intensity range (well below 50 μE photons) which largely avoids the formation of ROS and other stress related symptoms allowing to clearly separate low- and high-light effects on the electron transport chain (Mullineaux and Emlyn-Jones, 2005; Piippo et al., 2006; Wagner et al., 2008). It was shown in *Arabidopsis* that separate acclimation strategies to low and high-light conditions exist and that the plant is able to respond in quite different and distinct ways to these environmental signals (Bailey et al., 2001).

Knowledge obtained in such relatively simple experimental systems has been used to understand the much more complex situations in under realistic ecophysiological conditions. To this end recent studies investigated interactions of two or more parameters or the effects of permanently and/or freely fluctuating parameters to study the effects on the redox state of the electron transport chain and the corresponding acclimation responses (Kulheim et al., 2002; Frenkel et al., 2007; Tikkanen et al., 2010). Initial results strongly indicate that such variability in the environment creates a complex situation at the molecular level in the chloroplast which is difficult to interpret and to understand. Systems biology approaches in this field may help (see also below) as these have the power to integrate gene expression and metabolomics data (Bräutigam et al., 2009; Frenkel et al., 2009). However, bioinformatics and modeling needs to be further developed to be useful for future biological applications.

Finally, an important point which must be mentioned is that different species may respond in different ways in one and the same set-up simply because of their differing abilities, e.g., in the dissipation of excess excitation energy *via* non-photochemical quenching (NPQ). This process involves the action of the PsbS protein, a special member of the family of the light-harvesting complex proteins, and the activity of the xanthophyll cycle (Li et al., 2000; Niyogi et al., 2005). These contribute to a conformational change in the light harvesting complex of PSII under high light and the subsequent dissipation of absorbed excess light energy as heat counteracting the generation of ROS. The efficiency of NPQ, however, can be variable depending on the ecological specialization of the species (Demmig-Adams and Adams, 2006). Generalization from one species to other species, thus, is often difficult. However, comparative testing of various species or ecotypes in defined set-ups (now often called phenotyping) opens up the possibility to understand different ecological strategies of plant species and families which might help to engineer more stress-resistant or tolerant crops (Zhu et al., 2010). Development of a basic model with variable input and output parameters which puts redox signals into a framework of environmental acclimation responses in plants thus is highly desirable (Dietz and Pfannschmidt, 2011).

## Signal transduction of chloroplast redox signals toward the nucleus

The mechanisms by which redox signals from photosynthesis are transduced to the nucleus are largely not understood. Nevertheless, a number of proteins have been identified which are involved in the mediation of such signals at least within the plastid compartment.

The kinases STN7 and CSK1 are involved in the redox signaling from the PQ pool toward the plastid gene expression machinery which controls the adjustment of PS stoichiometry in response to long-term light quality shifts (Bonardi et al., 2005; Puthiyaveetil et al., 2008). Since PSs are composed also of nuclear encoded components this redox signaling requires a branch toward the nuclear compartment. Mutant analyses strongly suggest that the two signaling branches diverge at or directly after the STN7 kinase (Pesaresi et al., 2009). Further steps in this signaling cascade are unknown, but some experimental evidence exists that mediation of PQ redox signals both to plastid and nuclear gene expression machineries involve phosphorylation-dependent mechanisms (Escoubas et al., 1995; Steiner et al., 2009; Shimizu et al., 2010). As a theoretical possibility it has been discussed that PQ molecules from the thylakoid membrane system may also enter the envelope membrane of chloroplasts and directly signal the redox state to components associated or attached with it (Pfannschmidt et al., 2003). Experimental data for this are, however, lacking.

The transduction of redox signals from the thioredoxin system is mainly restricted to the understanding of SH-group-mediated control of enzymatic activities in the metabolism while it is not clear how this affects plastid gene expression. Existence of a thiol-dependent signal affecting chloroplast transcription beside the phosphorylation cascade mentioned above could be shown in *in organello* run-on transcription experiments (Steiner et al., 2009) and potential candidates for its transmission are the RNA polymerase subunits PAP6/FLN1 and PAP10/TrxZ (Arsova et al., 2010; Steiner et al., 2011) (Figure 1). The molecular connections, however, still require extensive further investigation.

Stress-induced hydrogen peroxide has been discussed to diffuse directly to the cytosol as it is the most stable ROS which can easily pass membranes (Pfannschmidt, 2003; Foyer and Noctor, 2005; Dietzel et al., 2008b). In the cytosol it can interact with a number of potential mediators including MAP kinase cascades or *rimb* (redox imbalanced) components (Kovtun et al., 2000; Heiber et al., 2007) which control down-stream redox regulators such as Rcd1 or Rap2.4a that affect nuclear gene expression (Shaikhali et al., 2008). ROS pattern have been also discussed as long-distance signals transmitted from cell to cell through the complete plant (Mittler et al., 2011). This involves signaling of high-light stress from exposed to unexposed leaves in *Arabidopsis* (Karpinski et al., 1999) as well as induction of cell death by singlet oxygen from PSII (Meskauskiene et al., 2001; Kim et al., 2012). The latter response can be genetically suppressed by inactivation of the chloroplast-located proteins Executer 1 (Ex1) and 2 (Ex2) indicating a role of them in transmission of singlet oxygen signals (Wagner et al., 2004). How this works mechanistically and which functions are exerted by Ex1 and Ex2 is unknown to date. Gun1 (*genomes uncoupled 1*) is a penta-tricopeptide repeat (PPR) protein with still unknown function which is postulated to merge plastidial signals from gene expression and ROS sending it to the nucleus (Cottage et al., 2007; Koussevitzky et al., 2007). This might involve the action of PTM, an envelope-localized PhD transcription factor which has been postulated to be released from plastids upon receiving a plastidial signal (possibly by Gun1) (Sun et al., 2011). After release from the outer membrane by a yet unknown protease it enters the nucleus and controls the expression of the ABI4 (*abscisic acid insensitive 4*) transcription factor. Finally, an indirect way of ROS signaling has been postulated to work *via* the ROS-sensitive glutathione biosynthesis pathway (so far only in *Arabidopsis*) since it appears that the synthesizing enzymes GSH1 and 2 are localized to the chloroplast and the cytosol, respectively. This would require the transport of γ-glutamyl-cysteine out of the chloroplast in order to synthesize the complete glutathione molecule in response to the accumulation of ROS (Mullineaux and Rausch, 2005; Wachter et al., 2005).

In conclusion, the effective transmission of plastidial redox signals both generated within the physiological range and under stress conditions is elusive although a number of interesting working hypotheses exist. How these models relate to each other, therefore, is a matter of future research.

## Redox-induced response patterns in nuclear gene expression

While the transmission of redox signals remained still elusive our understanding of the regulated target genes or gene groups has clearly improved in recent years. Initial evidence for effects of photosynthetic redox signals especially from the PQ pool on nuclear gene expression were obtained in unicellular algae (Escoubas et al., 1995; Maxwell et al., 1995) and, subsequently, also in vascular plants (Petracek et al., 1997; Pfannschmidt et al., 2001; Pursiheimo et al., 2001; Yang et al., 2001). This included changes in the transcript level, translation efficiency, or promoter usage. Typically, these set-ups used light treatments with combinations of electron transport inhibitors to affect the expression of nuclear encoded genes in model organisms like pea (Sullivan and Gray, 1999), tobacco (Pfannschmidt et al., 2001), or *Arabidopsis* (Fey et al., 2005). The favorite target gene in many cases was the Lhcb1 gene [encoding one major protein of the light-harvesting complex of PSII (LHCII)], the “classical” reporter for plastidial signaling since it displays a nicely regulated response pattern under different light treatments. In addition, also other photosynthesis-related genes were tested such as *PetE* (encoding plastocyanin), *Fed1* (encoding ferredoxin 1), or *Nia2* (encoding the cytosolic nitrate reductase) as well as the stress related *Apx2* gene (encoding the ascorbate peroxidase 2) (Karpinski et al., 1997; Petracek et al., 1997; Oswald et al., 2001; Sherameti et al., 2002). Nevertheless, all these reports must be regarded as pilot studies since they were restricted to a very small number of genes. Thus, they allowed the identification of signal origin and some regulation principles but were not representative for the response of specific gene groups such as photosynthesis associated nuclear genes (PhANGs) which are believed to be the primary target of plastidial signals.

With the successful sequencing of the *Arabidopsis* genome array technologies became available which allowed a genome-wide monitoring of nuclear gene expression changes in response to a variety of photosynthetic redox signals. This was first done with *Arabidopsis* plants which were subjected to a PQ reduction signal induced by a light quality shift from PSI- to PSII-light (Fey et al., 2005). In a parallel experiment plants were treated with the same light shift but the redox signal was blocked by the simultaneous application of DCMU. Redox controlled genes were identified by a comparison of the expression patterns in this set-up using a macroarray targeted to genes for proteins with predicted chloroplast location (Kurth et al., 2002). By this means 286 nuclear genes were identified to be under redox control. An additional inhibitor control, however, uncovered some unspecific side effect of the drug indicating that only 54 genes behaved in an “ideal” response pattern as expected if being under PQ redox control (Fey et al., 2005). Nevertheless, the number of regulated genes was unexpectedly high and not restricted to the expected target genes, i.e., PhANGs, but involved genes from all important gene groups such as gene regulation, signal transduction, or various biosynthetic pathways. In a different study *Arabidopsis* plants were subjected to light intensity and light quality shifts and the response pattern was detected with an array with around 8000 randomly selected *Arabidopsis* genes (Piippo et al., 2006). The general response was similar but it was concluded that the responsible redox signal was initiated in the stroma of chloroplasts not in the PQ pool.

In a more recent study an extensive kinetic approach was chosen to obtain a detailed picture of the dynamics of redox signaling and the corresponding response patterns. To this end *Arabidopsis* plants were subjected to either a reduction or an oxidation signal by using light-quality shifts and samples were taken directly before and 0.5, 2, 8, and 48 h after the shift (Bräutigam et al., 2009). The subsequent gene expression profiling revealed a number of important observations: (1) It demonstrated that the gene expression changes occurred in a very quick and dynamic manner indicating that a single observation time point (as done usually) reveals only a small part of the redox regulated genes. (2) It confirmed that metabolisms genes are a major part of the responsive gene groups and that PhANGs indeed display a unique response pattern among all regulated gene groups (Figure 1). (3) It indicated that redox signals from the PQ pool and stromal redox components are simultaneously active and a novel model for cooperative redox signaling was deduced (Bräutigam et al., 2010) which resolved the contradicting conclusion from the earlier studies mentioned above (Fey et al., 2005; Piippo et al., 2006).

A recent study used a genetic approach for studying redox responsive genes in *Arabidopsis* mutants with defects in the genes *stn7, psad1*, or *psae1* (encoding the thylakoid kinase STN7 and the PSI subunits PsaE1 and PsaD1) which are all devoid of state transitions and disturbed in redox signals from the PQ pool (Pesaresi et al., 2009). Comparison of transcript profiles of green-house grown plants led to the identification of 56 genes which were regulated in the same manner in all three genotypes representing either potential targets for PQ redox signals or putative representatives for a compensation response. Since in this study Affymetrix full-genome arrays were used also genes for non-chloroplast located proteins could be detected which were 39 out of the identified 56 genes, indicating that plastidial redox signals also affect genes for components in compartments other than plastids (Figure 1). This extends the potential influence of plastidial signals to the entire cell, demonstrating that this type of regulation is important not only for the plastid itself but appears to be a general part of the cellular signaling network (see also next chapter).

In an attempt to obtain a more detailed picture of the redox regulation network in *Arabidopsis* a systems biology study performed a meta-analysis of the kinetic and genetic approaches described above supplemented with further data from literature and data bases (Yao et al., 2011). Two transcription factors, ARR10 and ATH1-B, were proposed to be hubs in the redox gene regulatory network while the major photomorphogenesis regulator HY5 was considered to be not specifically affected in its connectivity by light-quality shifts and, thus, being not a specific component of the redox signaling network.

In summary, the recent array approaches revealed a much more complex redox regulation network in plant cells than originally anticipated. Apparently, photosynthetic redox signals do not only adjust photosynthesis genes but also genes coding for metabolic enzymes, signal transduction components, and gene regulation factors. This indicates a major role for redox signals in the cellular signaling networks of plants.

## Relation of redox- and photoreceptor-mediated light responses

**Figure 2:**
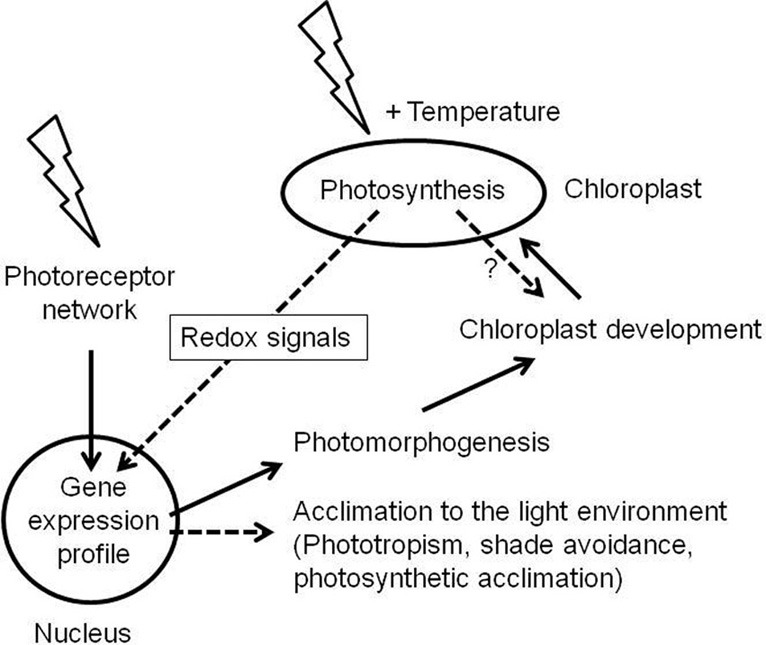
**Relation of photoreceptor- and photosynthesis-mediated light signaling.** Light (indicated by flash arrows) is sensed by photoreceptors in germinating and growing seedlings. They initiate gene expression profiles in the nucleus which run the photomorphogenic programme. A central part of this programme is the build-up of chloroplasts and the photosynthetic apparatus. As soon as this is functional it serves as additional light and temperature sensing system which affects nuclear gene expression by redox signals. Potential redox signals affecting chloroplast biogenesis are indicated by a question mark. Photoreceptor - and photosynthesis-mediated signals (solid and hatched black arrows, respectively) are integrated in the nucleus and induce modifying programmes which acclimate plant growth and function to the residing environment. The interconnectivity of the respective gene expression profiles is largely unknown; however, it is assumed that photoreceptors are dominant regulators of plant development while photosynthetic redox signals preferentially control acclimation responses.

Light is not only an energy source for plants but also provides important information which regulates major developmental responses such as photomorphogenesis of seedlings, shade avoidance responses, phototropism, circadian rhythms, or flower induction as well as more physiological responses such as chloroplast movement or stomatal opening (Jiao et al., 2007). These responses are regulated by a battery of photoreceptors which detect wavelengths and fluency rates of incident light such as the red/far-red light detecting phytochrome family (Smith, 1995), the blue light detecting cryptochromes and phototropins (Briggs and Christie, 2002; Lin and Shalitin, 2003), or the recently discovered UV-B light receptor (Rizzini et al., 2011). All these light-receptors control specific down-stream regulators which affect nuclear gene expression (Jiao et al., 2007). Since the photosynthetic light reaction can be driven with the same wavelengths it, thus, appears possible that there exists a potential cross-talk between the photoreceptor-mediated signaling networks and light-induced redox signals from photosynthesis. This assumption was initially supported by the observation that light and plastidial signals (induced by norflurazon treatment) act at the same *cis*-elements in the promoters of nuclear photosynthesis genes *Lhcb1* and *RbcS* (Kusnetsov et al., 1996; Strand et al., 2003; Acevedo-Hernandez et al., 2005). However, photoreceptor mutants revealed fully functional photosynthetic acclimation responses (Walters et al., 1999; Fey et al., 2005) indicating that photoreceptors are neither required nor essential for redox-controlled adjustment processes in chloroplasts. On the other hand it could be shown that a number of newly isolated cryptochrome 1 alleles behaved like weak *gun* alleles and it has been hypothesized that Gun1-mediated plastidial signals remodel light-signaling networks by interaction with the basic photomorphogenesis regulators Hy5 (Ruckle et al., 2007). This apparent contradiction can be resolved by identifying the class of plastidial signals being active in this context. Norflurazon treatment and *gun* mutants characterize a distinct class of plastid signals defined as “biogenic control” which represent signaling events essential for the proper build-up of the plastidial compartment (Pogson et al., 2008) and which are decisive especially in the very early phases of chloroplast generation (Pogson and Albrecht, 2011). In contrast, redox signals from photosynthesis become active only after photomorphogenesis was successfully performed, thus representing the most prominent class of signals defined as “operational control” (Pogson et al., 2008). Therefore, it can be hypothesized that photoreceptor-mediated signaling is dominant in morphological programmes which generate new tissues while photosynthetic redox signals become important only in existing tissues which must be functionally adjusted to the environment (Figure 2). New observations, however, suggest that this categorization is not as clear as assumed here. Studies in variegation mutants of *Arabidopsis* could demonstrate that the degree of variegation directly and positively correlates with the intensity of excitation pressure on the growing plant (Rosso et al., 2009). Very recent studies of chloroplast development in the shoot apex of *Arabidopsis* indicated that the fate of plastid development is determined in a very limited and small cell layer of the shoot apical meristem (Charuvi et al., 2012) providing a morphological indication that supports the likeliness of the excitation pressure model. Furthermore, other results suggest a link between plant resistance responses and plastids which might be light-mediated (Ballare et al., 2012). It appears that plastidial signals can modify plant defence responses although the molecular links are not understood yet (Karpinski et al., 2003; Kangasjarvi et al., 2012). The strong vertical light quality gradients within dense plant populations affect both the phytochrome system *via* the red to far-red ratio as well as the excitation energy distribution between the PSs. A parallel action of both signaling networks thus would be conceivable. Array data obtained in set-ups investigating light-quality regulated genes, however, uncovered only very small overlap between phytochrome- and redox-mediated transcript profiles (Bräutigam et al., 2009). This argues for a parallel rather than an interacting influence on nuclear gene expression; however, to provide final proof for this conclusion array experiments which directly address this specific question needs to be designed and performed.

In conclusion, we need more knowledge to understand how the two fundamental light-dependent signaling networks, controlled by photoreceptors and photosynthetic redox signals, are integrated to regulate nuclear gene expression. As another complication one also needs to consider the action of mitochondria, which are tightly connected to chloroplast function and redox state (Raghavendra and Padmasree, 2003). Future work needs to integrate communication pathways and metabolic interaction of the three different genetic compartments of plant cells in order to obtain a comprehensive view how they respond to environmental constraints in a coordinated manner (Pfannschmidt, 2010).

### Conflict of interest statement

The authors declare that the research was conducted in the absence of any commercial or financial relationships that could be construed as a potential conflict of interest.
